# Supplementary material for: Meteorin-like is an injectable peptide that can enhance regeneration in aged muscle through immune-driven fibro/adipogenic progenitor signaling
Source: Nat Commun. 2022 Dec 9;13:7613. doi: 10.1038/s41467-022-35390-3 (PMC9734561; doi:10.1038/s41467-022-35390-3)
Supplement: Supplementary file 1 — Supplementary Information [file 41467_2022_35390_MOESM1_ESM.pdf]

Title: Meteorin-like is an injectable peptide that can enhance regeneration in aged muscle through immune-driven fibro/adipogenic progenitor signaling

Author List: David E. Lee[1,2], Lauren K. McKay[2,3], Akshay Bareja[1,2], Yongwu Li[4], Alastair Khodabukus[4], Nenad Bursac[4], Gregory A. Taylor[5-9], Gurpreet S. Baht[2,10], and James P. White[1,2,9*]

Figure S1

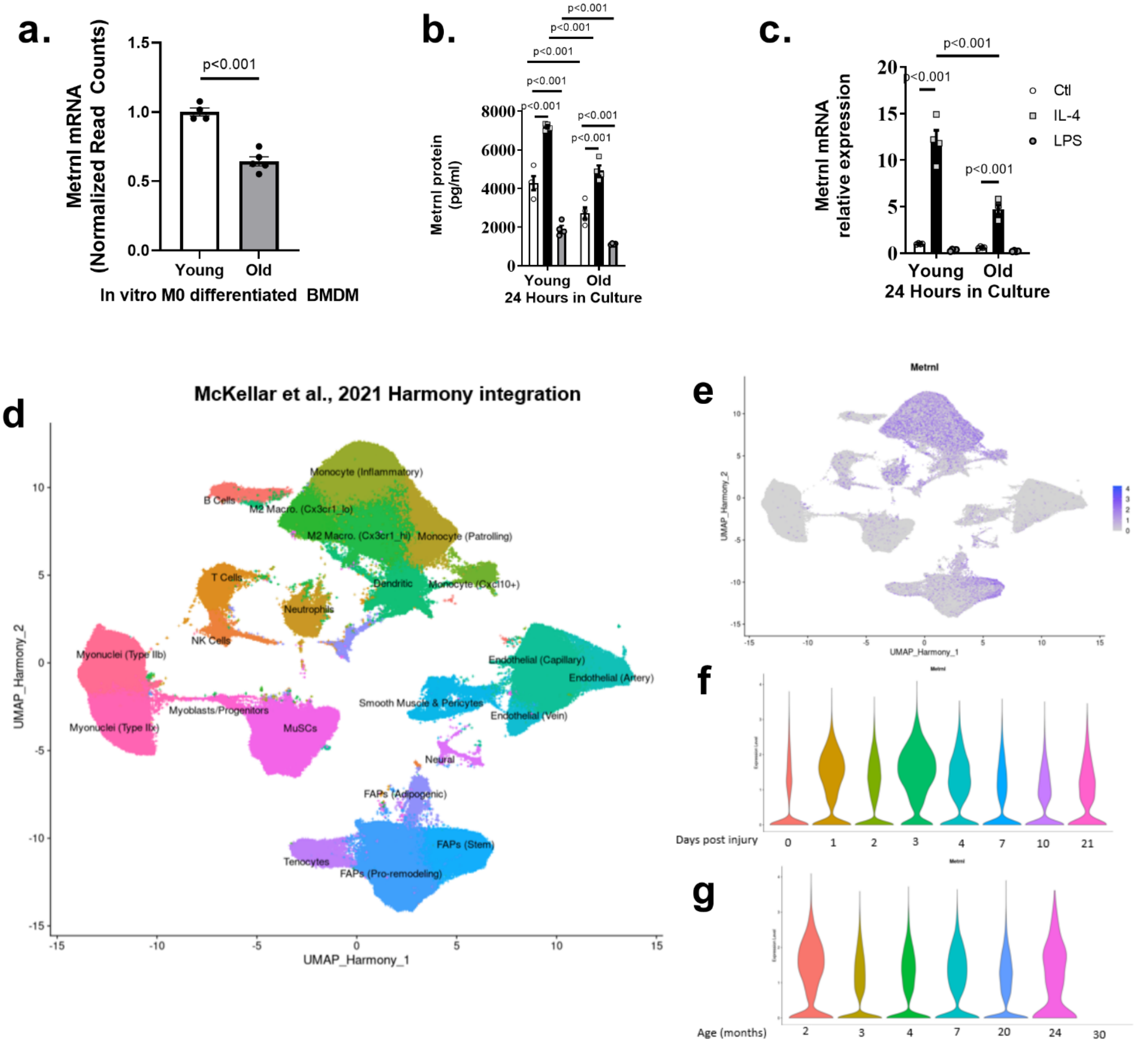

Supplemental Figure 1. Supplemental data to Figure 2. A) mRNA from cells were analyzed by RNAseq – following data normalization results of *Metrnl* gene expression are presented; n = 3 animals/group; two-tailed t-test was performed. B) METRNL protein measured in the conditioned media and C) mRNA of cells from experiments on primary monocytes isolated from bone marrow and cultured in MCSF (Ctrl), IL-4 or LPS to induce macrophage differentiation (n=4 animals/group); 2x3 ANOVA with Fisher's LSD post hoc was used for analysis. D) UMAP clustering and identifications of Harmony integration scRNA-seq analysis performed and previously published by McKellar et al. (PMID 34773081) which includes >365,000 cells isolation from skeletal muscle under various states of muscle injury and mouse age. E) Featureplot of UMAP with expression of Metrnl overlaid by cell. Violin Plots of Metrnl expression frequency from immune cell clusters split by f) injury timepoint and g) age of mouse used for analysis. p value is indicated with connecting line between comparison groups. Source Data are provided as a source data file. Data are presented as mean values +/- SEM.

Figure S2

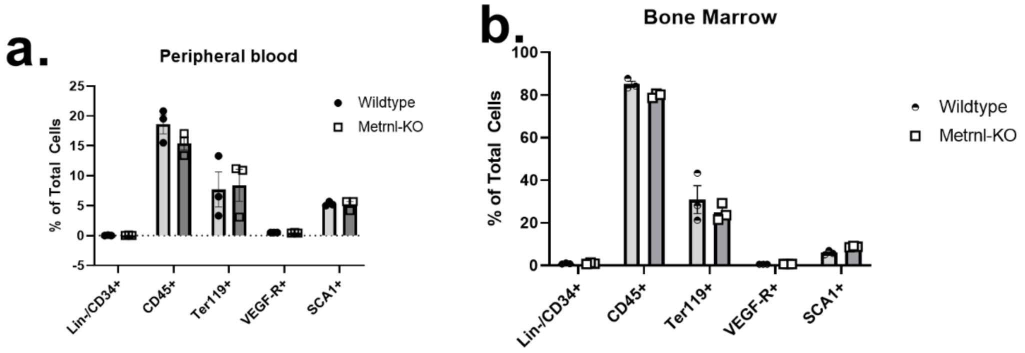

Supplemental Figure 2. Whole peripheral blood (a) and bone marrow (b) were analyzed from wild-type and Metrnl(-/-) mice by flow cytometry to proportions in cell populations (n=3/group); two-tailed t-test was performed. p value is indicated with connecting line between comparison groups if significantly different. Source Data are provided as a source data file. Data are presented as mean values +/- SEM.

Figure S3

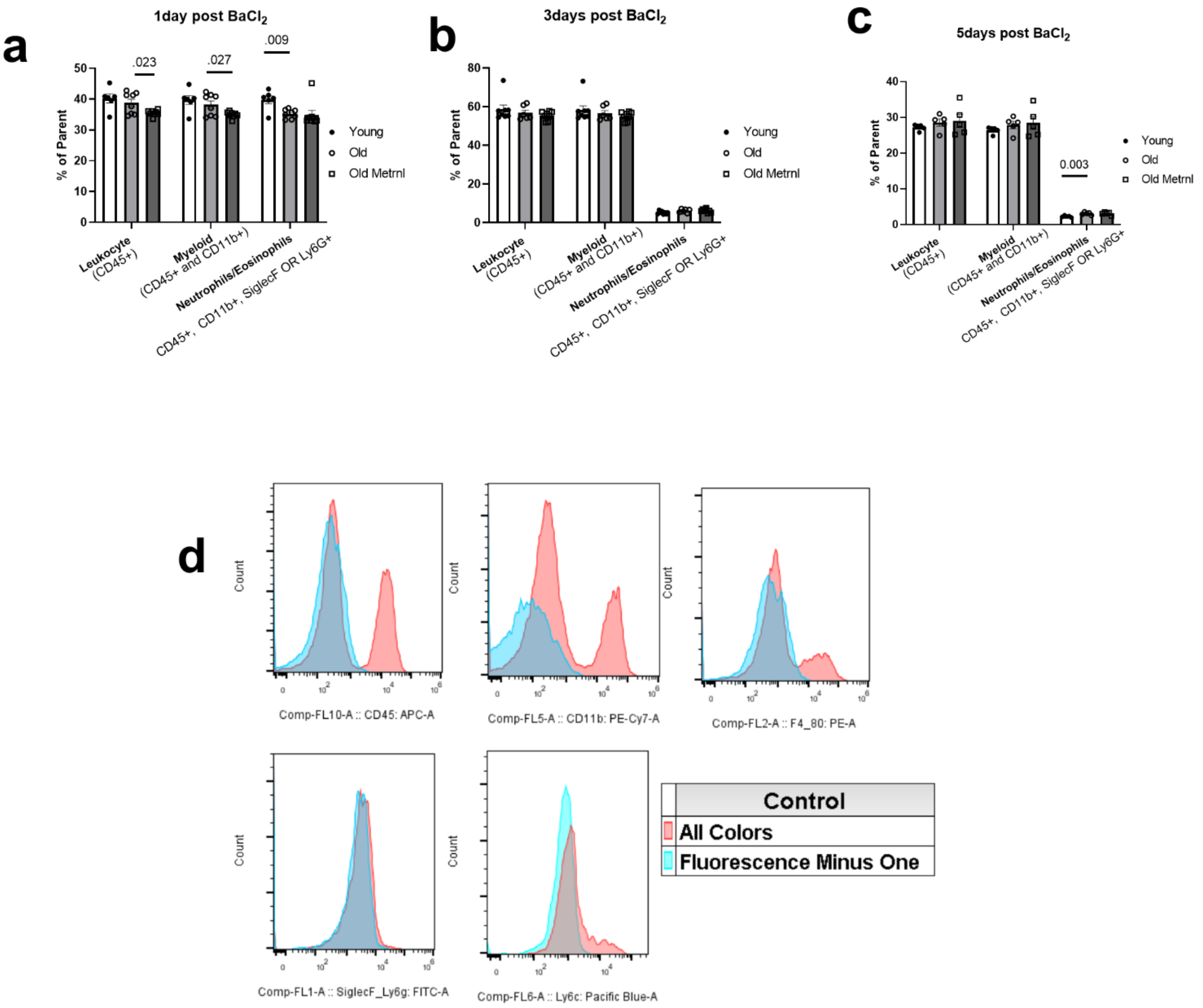

Supplemental Figure 3. Supplement to Figure 3B-E. In figure 3C-D, data are shown regarding cell subpopulations gated using strategy shown in Figure 3B. The quantification of parent populations including leukocytes (singlets gated as CD45+), Myeloid cells (leukocytes gated as CD11b+), and a population that combined neutrophil and eosinophil markers (SiglecF and Ly6G) on a single fluorescent channel as a proportion of total myeloid cells is shown for: (A) 1 day (6 young, 8 old, 8 old + Metrnl), (B) 3 days (n = 8 young, 6 old, 8 old+rMetrnl), and (C) 5days (n = 5/group) post $BaCl_2$ injury; One-way ANOVA was used with Fisher's LSD posthoc analysis at each timepoint. D) Example histograms of each fluorescence minus one control and all color stained control used to establish flow cytometry gating. p value is indicated with connecting line between comparison groups. Source Data are provided as a source data file. Data are presented as mean values +/- SEM.

Figure S4

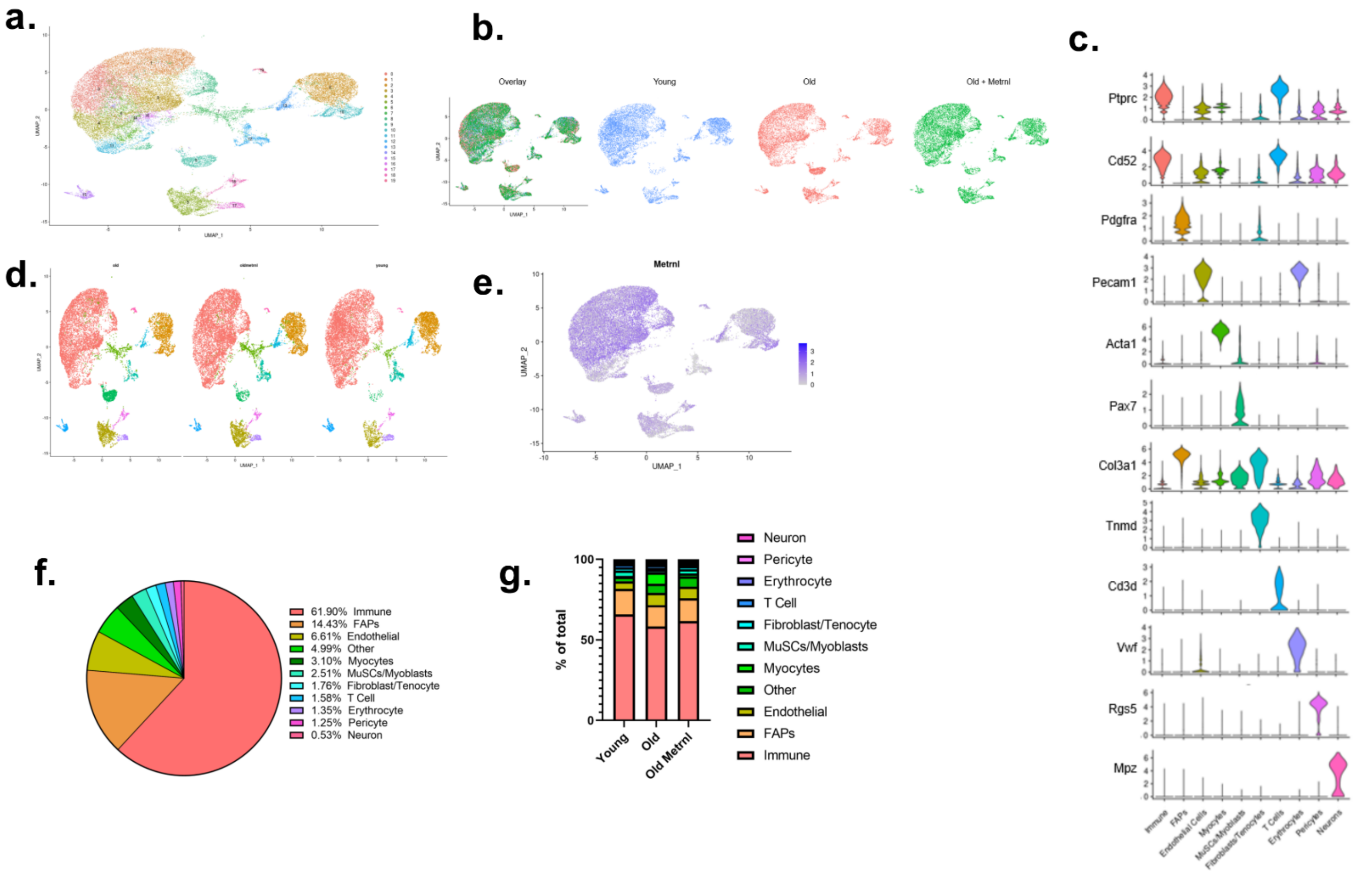

Supplemental Figure 4. Figure supplement to Figure 5. A) initial cell groupings identified by UMAP dimensional reduction (20 distinguished clusters). B) Overlaid and separated UMAP reductions show similar coverage across all cell types from each sample grouping. C) Violin plot showing uniquely enriched gene markers for each cell identified cell cluster. D) Same as B with colors depicting final cell classifications from C. E) UMAP reduction with feature scaling of all cells based on *Metrnl* expression levels over represented by immune cells. F,G) Percent of cells from each cell classification.

Figure S5

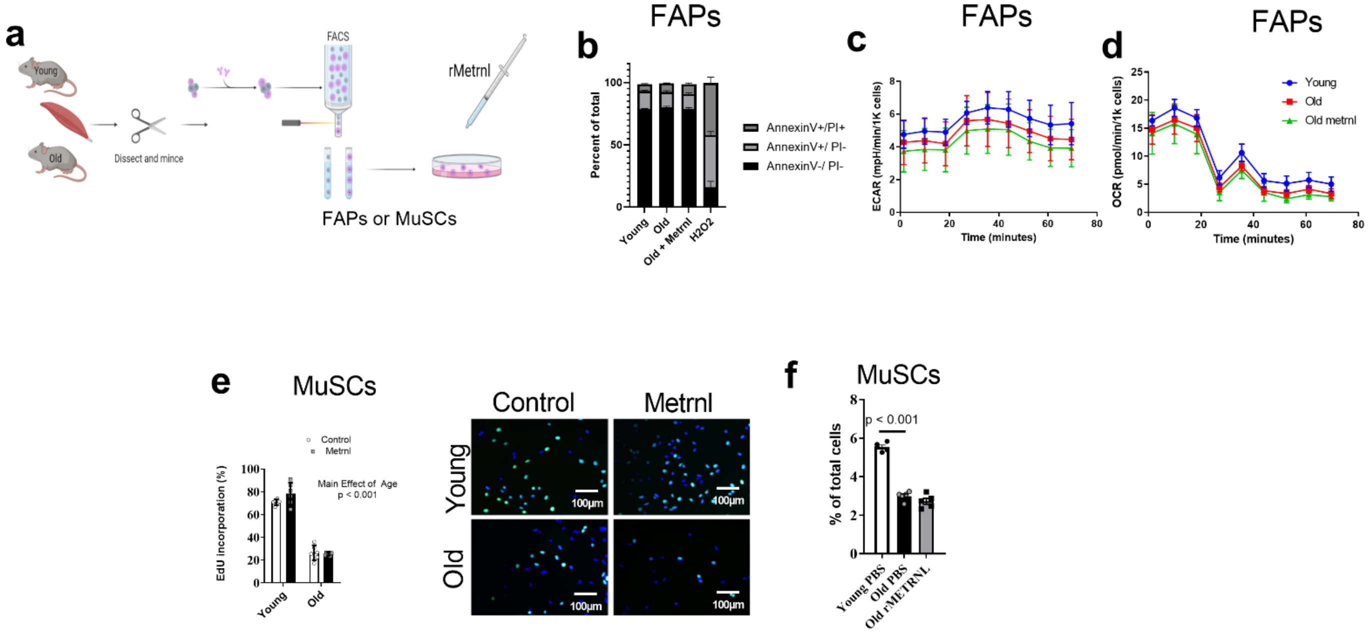

Supplemental Figure 5. Metrnl treatment directly on FAPs, MuSCs or c2c12 myotubes does not result in any significant changes to proliferative capacity, survival, or metabolic phenotype. A) shows experimental strategy of direct treatment with rMETRNL on isolated cells. B) Annexin staining of FAPs (n = 3 animals/group); One-way ANOVA was used with Fisher's LSD posthoc analysis. C,D) Extracellular flux analysis of FAPs using Seahorse Flux analysis (n = 4 young, 3 old, 3 old +rMetrnl) One-way ANOVA was used with Fisher's LSD posthoc analysis at each timepoint. E) Isolated MuSCs were assessed for proliferative capacity ex vivo (n = 6/group); One-way ANOVA was used with Fisher's LSD posthoc analysis. F) MuSCs were quantified by flow cytometry of VCAM+ cells following $BaCl_2$ injury (n = 5/group); One-way ANOVA was used with Fisher's LSD posthoc analysis. p value is indicated with connecting line between comparison groups. Source Data are provided as a source data file. Graphics created with BioRender.com. Data are presented as mean values +/- SEM.

Figure S6

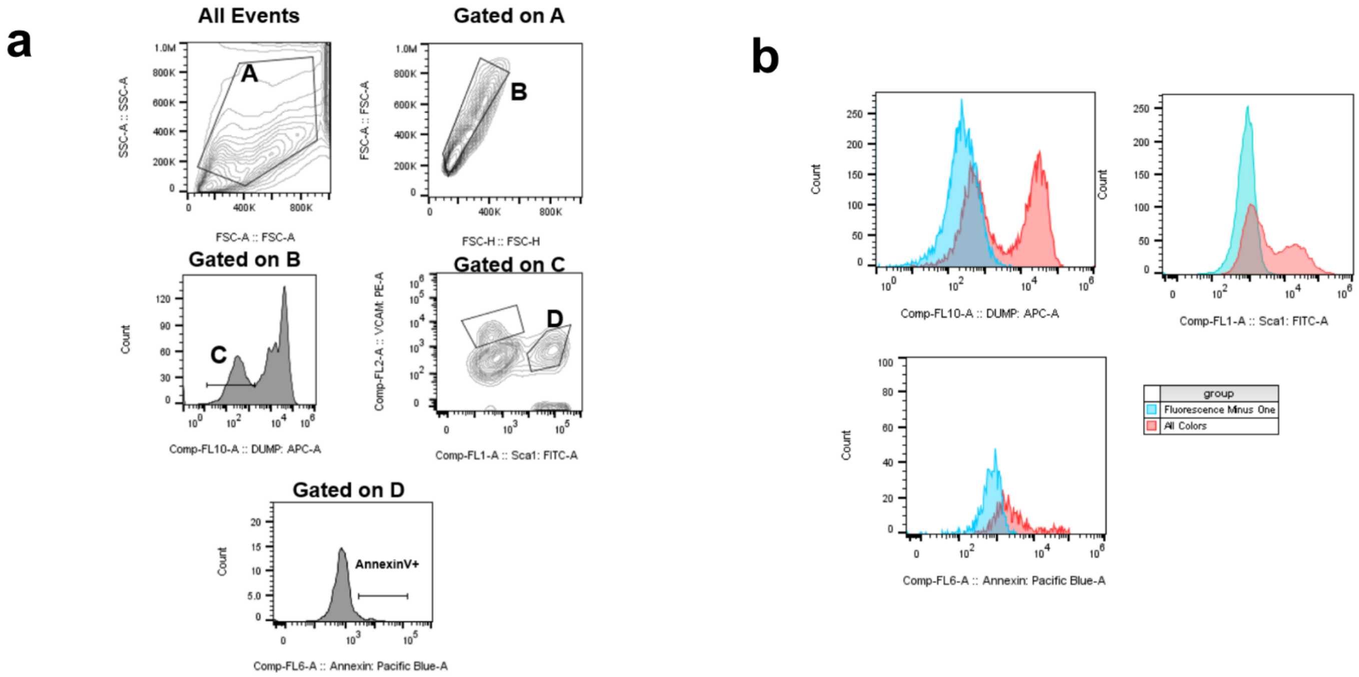

Supplemental Figure 6. Supplement to Figure 6. A) Gating strategy used for flow cytometry of Sca1+ FAPs and annexin V staining. B) Fluorescence Minus one controls used for setting gates in A.

Supplemental Figure 7

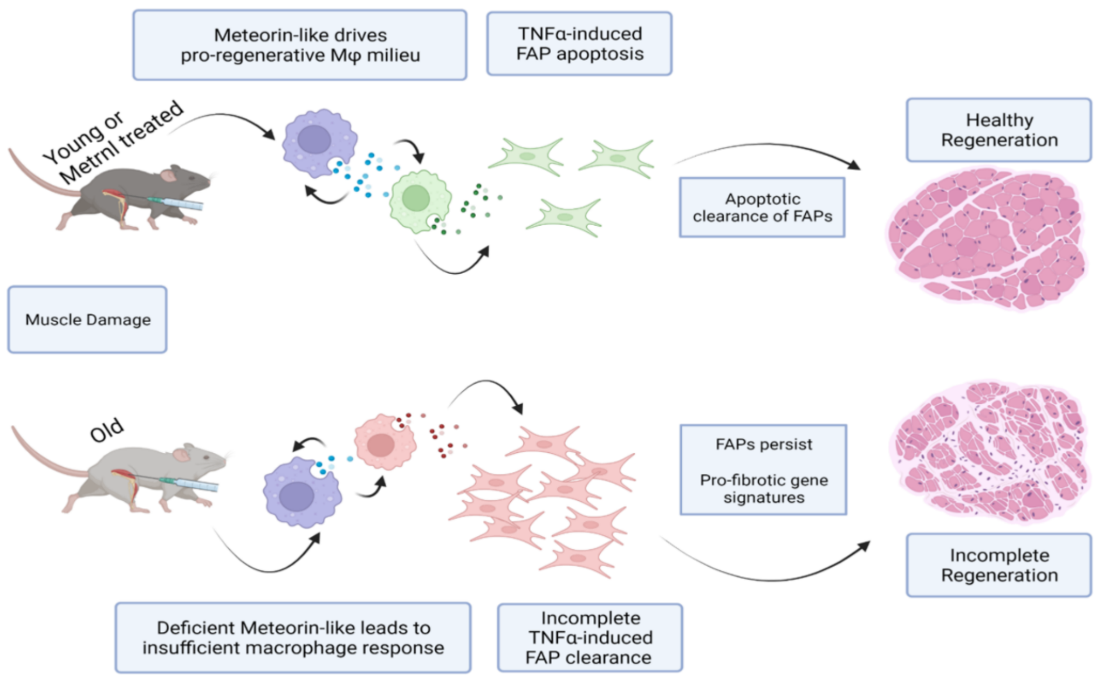

Supplemental Figure 7. Graphical representation of main conclusions. Graphics created with BioRender.com.

Supplemental Table 1. Primers used for SYBR-based RT qPCR

Gene	forward	reverse
col1a1	CGATGGATTCCCGTTCGAGT	GAGGCCTCGGTGGACATTAG
fn1	GACCCTTACACGGTTTCCCA	TGGCACCATTTAGATGAATCGC
pdgfra	GCAGTTGCCTTACGACTCCAGA	GGTTTGAGCATCTTCACAGCCAC
18s	GGACCAGAGCGAAAGCATTTGCC	TCAATCTCGGGTGGCTGAACGC
metrnl	CTGGAGCAGGGAGGCTTATTT	GGACAACAAAGTCACTGGTACAG
