# Supplementary material for: Meteorin-like is an injectable peptide that can enhance regeneration in aged muscle through immune-driven fibro/adipogenic progenitor signaling
Source: Nat Commun. 2022 Dec 9;13:7613. doi: 10.1038/s41467-022-35390-3 (PMC9734561; doi:10.1038/s41467-022-35390-3)
Supplement: Supplementary file 3 — Description of Additional Supplementary files [file 41467_2022_35390_MOESM3_ESM.pdf]

Description of Additional Supplementary Files

Supplementary Data 1. Results of RNA sequencing data related to Supplementary figure 1a.
