# Supplementary material for: Meteorin-like is an injectable peptide that can enhance regeneration in aged muscle through immune-driven fibro/adipogenic progenitor signaling
Source: Nat Commun. 2022 Dec 9;13:7613. doi: 10.1038/s41467-022-35390-3 (PMC9734561; doi:10.1038/s41467-022-35390-3)
Supplement: Supplementary file 5 — Reporting Summary [file 41467_2022_35390_MOESM5_ESM.pdf]

nature portfolio

Corresponding author(s): James P White

Last updated by author(s): Nov 3, 2022

Reporting Summary

Nature Portfolio wishes to improve the reproducibility of the work that we publish. This form provides structure for consistency and transparency in reporting. For further information on Nature Portfolio policies, see our Editorial Policies and the Editorial Policy Checklist.

Statistics

For all statistical analyses, confirm that the following items are present in the figure legend, table legend, main text, or Methods section.

n/a	Confirmed	
☐	☒	The exact sample size (*n*) for each experimental group/condition, given as a discrete number and unit of measurement
☐	☒	A statement on whether measurements were taken from distinct samples or whether the same sample was measured repeatedly
☐	☒	The statistical test(s) used AND whether they are one- or two-sided<br>*Only common tests should be described solely by name; describe more complex techniques in the Methods section.*
☒	☐	A description of all covariates tested
☐	☒	A description of any assumptions or corrections, such as tests of normality and adjustment for multiple comparisons
☐	☒	A full description of the statistical parameters including central tendency (e.g. means) or other basic estimates (e.g. regression coefficient) AND variation (e.g. standard deviation) or associated estimates of uncertainty (e.g. confidence intervals)
☐	☒	For null hypothesis testing, the test statistic (e.g. *F*, *t*, *r*) with confidence intervals, effect sizes, degrees of freedom and *P* value noted<br>*Give P values as exact values whenever suitable.*
☒	☐	For Bayesian analysis, information on the choice of priors and Markov chain Monte Carlo settings
☒	☐	For hierarchical and complex designs, identification of the appropriate level for tests and full reporting of outcomes
☒	☐	Estimates of effect sizes (e.g. Cohen's *d*, Pearson's *r*), indicating how they were calculated

Our web collection on statistics for biologists contains articles on many of the points above.

Software and code

Policy information about availability of computer code

Data collection	No custom scripts or code were used for data collection.
Data analysis	Scripts were used for data analysis using the R studio (V4.2) and functions associated with Seurat (V4.0) and related dependencies. Raw scRNA-seq data were analyzed using CellRanger software (V5.0.0). FlowJo (V10.8) was used for flow cytometry data processing.

For manuscripts utilizing custom algorithms or software that are central to the research but not yet described in published literature, software must be made available to editors and reviewers. We strongly encourage code deposition in a community repository (e.g. GitHub). See the Nature Portfolio guidelines for submitting code & software for further information.

Data

Policy information about availability of data

All manuscripts must include a data availability statement. This statement should provide the following information, where applicable:

- Accession codes, unique identifiers, or web links for publicly available datasets
- A description of any restrictions on data availability
- For clinical datasets or third party data, please ensure that the statement adheres to our policy

Data availability statement is included in the manuscript text. Whole muscle mRNA sequencing data (Fig 4) has been deposited to GEO at number: GSE217037 (https://www.ncbi.nlm.nih.gov/geo/query/acc.cgi?acc=GSE217037). Single Cell RNA sequencing data (Fig 5) has been deposited to GEO at number: GSE217258

(https://www.ncbi.nlm.nih.gov/geo/query/acc.cgi?acc=GSE217258) . All other related is available in accompanying source data files. Reactome database used can be accessed via reactome.org. Gene Ontology database can be accessed via geneontology.org.

Human research participants

Policy information about studies involving human research participants and Sex and Gender in Research.

Reporting on sex and gender	N/A
Population characteristics	N/A
Recruitment	N/A
Ethics oversight	N/A

Note that full information on the approval of the study protocol must also be provided in the manuscript.

Field-specific reporting

Please select the one below that is the best fit for your research. If you are not sure, read the appropriate sections before making your selection.

☒ Life sciences ☐ Behavioural & social sciences ☐ Ecological, evolutionary & environmental sciences

For a reference copy of the document with all sections, see nature.com/documents/nr-reporting-summary-flat.pdf

Life sciences study design

All studies must disclose on these points even when the disclosure is negative.

Sample size	All animal study n's were determined by a prior power analyses to determine adequate sample sizes to achieve minimum B=0.80.
Data exclusions	No data were excluded from analyses once samples were collected. Rarely (<5%) mice died prematurely following BaCl2 injections so could not reach experimental endpoints.
Replication	All animal studies were performed using 3-8 biological replicates/individual mice (exact n noted in figure legends). For qPCR analyses, each biological replicate was analyzed in technical triplicate. For tissue culture, each experiment was repeated in three independent replicates. For flow cytometry, each biological sample was analyzed for minimum 10,000 singlet gated cells. All attempts at replication experiments were successful.
Randomization	All young mice were used as controls. All old mice were randomly distributed between old-control and old-experimental groups prior to the onset of experimentation. For heterochronic parabiosis, mice were prescreened by animal surgeon to best match body sizes of paired mice.
Blinding	Samples were coded upon collection through analysis and decoded after quantitation for flow cytometry, force measurements and molecular assays. For histological analysis, images were coded and quantified either by blinded technician or unbias macro script.

Reporting for specific materials, systems and methods

We require information from authors about some types of materials, experimental systems and methods used in many studies. Here, indicate whether each material, system or method listed is relevant to your study. If you are not sure if a list item applies to your research, read the appropriate section before selecting a response.

Materials & experimental systems

n/a	Involved in the study	
☐	☒	Antibodies
☒	☐	Eukaryotic cell lines
☒	☐	Palaeontology and archaeology
☐	☒	Animals and other organisms
☒	☐	Clinical data
☒	☐	Dual use research of concern

Methods

n/a	Involved in the study	
☒	☐	ChIP-seq
☐	☒	Flow cytometry
☒	☐	MRI-based neuroimaging

Antibodies

Antibodies used	Antibodies used include the following: APC anti-mouse CD45 (1:200, Biolegend 157605), APC anti-mouse CD11b (1:200, Biolegend, 101211), PE anti-mouse CD106 (1:50, VCAM, Biolegend 105713), FITC anti-mouse Ly6A/E (SCA1, 1:50, Biolegend, 108105), Pacific Blue anti-mouse Ly6C (1:200, Biolegend, 128013), AlexaFluoro 488 anti-mouse CD170 (SiglecF, 1:200, Biolegend 155524), AlexaFluoro 488 anti-mouse Ly6G (1:200, Biolegend 127626), APC anti mouse F4/80 (1:200, Biolegend 123116), Pacific Blue AnnexinV (Biolegend 640926), PE-Cy7 anti-mouse CD11b (1:200, Biolegend 101216).
Validation	All antibodies were validated by manufacturer and each lot is quality control tested for flow cytometric analysis. From manufacturer: "Each lot product is validated by QC testing with a series of titration dilutions."

Animals and other research organisms

Policy information about studies involving animals; ARRIVE guidelines recommended for reporting animal research, and Sex and Gender in Research

Laboratory animals	mus musculus (C57/bl6j) mice were used for these experiments. Mice were provided by NIA and used for young groups at 3-4months age and used for old grouping at 24-27 months age. 3-4 month old Metrnl-/- mice (similar B56 background) were used for transgenic experiments as described in the animal methods.
Wild animals	No wild animals were used in this study.
Reporting on sex	The response to muscle injury in expression of Metrnl mRNA was no different in male vs female mice. We maintained sex as a control throughout most of the experimentation (males) with the exception for heterochronic parabiosis studies which used only females in order to reduce fighting after surgical pairing.
Field-collected samples	No field-collected samples were used in this study.
Ethics oversight	All animal experiments and care followed the guidelines and were approved by the Institutional Animal Care and Use Committee at Duke Medical Center.

Note that full information on the approval of the study protocol must also be provided in the manuscript.

Flow Cytometry

Plots

Confirm that:

☒ The axis labels state the marker and fluorochrome used (e.g. CD4-FITC).

☒ The axis scales are clearly visible. Include numbers along axes only for bottom left plot of group (a 'group' is an analysis of identical markers).

☒ All plots are contour plots with outliers or pseudocolor plots.

☒ A numerical value for number of cells or percentage (with statistics) is provided.

Methodology

Sample preparation	The majority of the flow cytometry experiments were performed on mouse tibialis anterior muscles that were dissected, digested using collagenase solution, filtered, stained with antibodies and analyzed by flow cytometry.
Instrument	Sony MA900
Software	Sony MA900 cell sorting software was used for initial analysis and data collection. data were exported as FCS 3.0 files and analyzed using FlowJo software.
Cell population abundance	When cell sorting was performed, a post sort analysis was performed on ~100 cells to verify >95% purity of sorted populations).
Gating strategy	Gating strategy for all flow cytometry began by identifying cells using FSC-A and BSC-A channels and then FSC-H by FSC-A correlation. Specific Gating strategy for macrophage populations is displayed in Figure 3B. Specific gating strategy for FAPs is displayed in Supplemental figure 5 A. Fluorescence minus one controls were used to set gates. Fluorescence minus one controls for each gating strategy are shown in supplemental figures.

☒ Tick this box to confirm that a figure exemplifying the gating strategy is provided in the Supplementary Information.
